# The Impact of Preoperative Patient Education on Postoperative Pain, Opioid Use, and Psychological Outcomes: A Narrative Review

**DOI:** 10.1080/24740527.2023.2266751

**Published:** 2023-11-28

**Authors:** Rasheeda Darville-Beneby, Anna M. Lomanowska, Hai Chuan Yu, Parker Jobin, Brittany N. Rosenbloom, Gretchen Gabriel, Helena Daudt, Michael Negraeff, Tania Di Renna, Maria Hudspith, Hance Clarke

**Affiliations:** a Department of Anesthesiology and Pain Medicine, University of Toronto, Toronto, Ontario, Canada; b Department of Anesthesia and Pain Management Pain Research Unit, Toronto General Hospital, Toronto, Ontario, Canada; c Transitional Pain Service, Toronto General Hospital, Toronto, Ontario, Canada; d Department of Anesthesiology, Perioperative and Pain Medicine, University of Calgary, Calgary, Alberta, Canada; e Department of Medicine, Cedars Sinai Medical Center, Los Angeles, California, USA; f Child Health Evaluative Sciences, the Hospital for Sick Children, Toronto, Ontario, Canada; g Pain BC/Pain Canada, Vancouver, British Columbia, Canada; h Vancouver General Hospital, Vancouver, British Columbia, Canada; i Toronto Academic Pain Medicine Institute, Toronto, Ontario, Canada

**Keywords:** preoperative, patient education, elective surgery

## Abstract

**Background:**

Recent studies have shown that preoperative education can positively impact postoperative recovery, improving postoperative pain management and patient satisfaction. Gaps in preoperative education regarding postoperative pain and opioid use may lead to increased patient anxiety and persistent postoperative opioid use.

**Objectives:**

The objective of this narrative review was to identify, examine, and summarize the available evidence on the use and effectiveness of preoperative educational interventions with respect to postoperative outcomes.

**Method:**

The current narrative review focused on studies that assessed the impact of preoperative educational interventions on postoperative pain, opioid use, and psychological outcomes. The search strategy used concept blocks including “preoperative” AND “patient education” AND “elective surgery,” limited to the English language, humans, and adults, using the MEDLINE ALL database. Studies reporting on preoperative educational interventions that included postoperative outcomes were included. Studies reporting on enhanced recovery after surgery protocols were excluded.

**Results:**

From a total of 761 retrieved articles, 721 were screened in full and 34 met criteria for inclusion. Of 12 studies that assessed the impact of preoperative educational interventions on postoperative pain, 5 reported a benefit for pain reduction. Eight studies examined postoperative opioid use, and all found a significant reduction in opioid consumption after preoperative education. Twenty-four studies reported on postoperative psychological outcomes, and 20 of these showed benefits of preoperative education, especially on postoperative anxiety.

**Conclusion:**

Preoperative patient education interventions demonstrate promise for improving postoperative outcomes. Preoperative education programs should become a prerequisite and an available resource for all patients undergoing elective surgery.

## RÉSUMÉ

**Contexte:** Des études récentes ont démontré que l’éducation préopératoire peut avoir un effet positif sur la récupération postopératoire en améliorant la prise en charge de la douleur postopératoire et la satisfaction des patients. Des lacunes dans l’éducation préopératoire concernant la douleur postopératoire et l’utilisation d’opioïdes peuvent entraîner une augmentation de l’anxiété chez les patients et une utilisation persistante d’opioïdes après l’opération.

**Objectifs:** L’objectif de cette revue narrative était de recenser, d’examiner et de résumer les données probantes disponibles sur l’utilisation et l’efficacité des interventions éducatives préopératoires en ce qui concerne les résultats postopératoires.

**Méthode:** Cette revue narrative s’est concentrée sur des études évaluant l’effet des interventions éducatives préopératoires sur la douleur postopératoire, l’utilisation d’opioïdes et les résultats psychologiques. La stratégie de recherche a eu recours à des blocs de concepts comprenant « préopératoire » ET « éducation des patients » ET « chirurgie élective », limités à la langue anglaise, aux humains et aux adultes, en utilisant la base de données MEDLINE ALL. Les études portant sur des interventions éducatives préopératoires qui comprenaient des résultats postopératoires ont été incluses, tandis que celles qui décrivaient une amélioration de la récupération après des interventions chirurgi ont été exclues.

**Résultats:** Sur un total de 761 articles recensés, 721 ont été examinés en entier et 34 répondaient aux critères d’inclusion. Parmi les 12 études évaluant l’effet des interventions éducatives préopératoires sur la douleur postopératoire, cinq ont rapporté des avantages pour la diminution de la douleur. Huit études ont examiné l’utilisation d’opioïdes postopératoires, et toutes ont constaté une diminution significative de la consommation d’opioïdes suite à une éducation préopératoire. Vingt-quatre études ont rendu compte des résultats psychologiques postopératoires, et vingt d’entre elles ont démontré que l’éducation préopératoire présentait des avantages, en particulier en ce qui concerne l’anxiété postopératoire.

**Conclusion:** Les interventions éducatives préopératoires présentent des perspectives prometteuses pour améliorer les résultats postopératoires. Les programmes d’éducation préopératoire devraient devenir une condition préalable et une ressource disponible pour tous les patients subissant une chirurgie élective.

## Introduction

Acute pain after a surgical procedure is routinely treated with short-term opioid medication.^1^ For many patients, however, acute pain after surgery can progress to become chronic.^2^ Referred to as chronic postsurgical pain, this condition has a median incidence of 20% to 30% during the 6 to 12 months after surgery.^3,4^ Chronic postsurgical pain can lead to reduced function and quality of life,^5,6^ as well as persistent opioid use after surgery and an increased risk of opioid use disorder and overdose.^7,8^ Targeted interventions during the perioperative period offer an opportunity to prevent the progression from acute to chronic pain and subsequent negative consequences for functioning and persistent opioid use.^7,9–11^ In particular, there is interest in the benefits of preoperative education for postoperative recovery to help patients manage expectations about pain after surgery and to promote safe prescription opioid use.^12–14^ This narrative review provides an overview of the available evidence on the use and effectiveness of preoperative education to inform future interventions aimed at improving postoperative pain management and safe opioid use.

### Prescription Opioid Use and the Opioid Epidemic

According to the Canadian Medical Protection Association, more than 1 million surgical procedures are performed annually in Canada.^15^ Opioids are a class of powerful analgesic medications that are routinely prescribed for postoperative pain management, and surgery remains the most common indication for opioid initiation.^1^ However, the persistent use of prescription opioids after surgery can lead to opioid use disorder and has been considered as a contributing factor to the ongoing opioid crisis.^16,17^ According to the Centers for Disease Control and Prevention, having a history of a prescription for an opioid pain medication increases the risk of overdose and opioid use disorder.^18^ Similarly, excessive postoperative opioid use increases the risk of drug diversion and the development of persistent opioid use with the possibility of developing an opioid use disorder and overdose.^1,17^

Opioid overdose continues to be a major cause of mortality in Canada. According to Health Canada, there were a total of 36,442 apparent opioid toxicity deaths between January 2016 and December 2022.^19^ Between 2016 and 2018, more than 9000 Canadians died from apparent opioid-related harms.^20^ Though most deaths have been due to illicit opioid use, prescription opioids continue to be a focus for public health reform^21^ because chronic opioid treatment has been linked to increased likelihood of illicit drug use.^22,23^ For instance, in a sample of individuals in British Columbia who experienced an overdose between 2015 and 2016, around half of the sample had been prescribed opioids for pain in the previous 5 years, but most did not have an active opioid prescription at the time of overdose.^23^ Addressing persistent pain after surgery and related persistent opioid use is an important avenue to reducing later problematic opioid use.

### Initiatives to Minimize Postoperative Opioid Consumption

The Canadian Institute for Health Information stated that in 2018 almost one in eight people in the study population were prescribed opioids.^21^ In Canada, consumption of prescription opioids increased 202.8% between 2001 to 2009, with a further 6.4% increase observed from 2010 to 2015.^21^ In light of this considerable increase, the Canadian physician community responded by reducing opioid prescribing. In subsequent years, from 2013 to 2018, the proportion of people prescribed opioids decreased from 14.3% to 12.3%, representing an overall 8% decrease in the number of persons taking opioids.^21^

Other initiatives have also been established to minimize postoperative opioid consumption and misuse, including evidence-based prescribing guidelines.^24^ The higher the opioid dose, the higher the risk for misuse and overdose^25^ and the lower the likelihood that a patient is able to wean from their opioid medication postdischarge.^26^ These risks further increase in patients with an active or prior substance use disorder and concurrent psychological diagnoses.^26^ There is evidence to suggest that an overdose risk exists at doses as low as <20 mg morphine equivalents daily (MED).^27^ A significant increase in overdose risk occurs if patients consume greater than 50 mg MED.^28^ Current guidelines regarding safe opioid prescribing practices suggest that patients commencing opioid therapy be restricted to <50 MED, with the maximum prescribed dose to <90 MED.^27^ However, in the perioperative setting, patients are often discharged with doses exceeding these recommendations.^26^ In addition to prescribing guidelines, programs that can decrease the use of postoperative opioids by educating patients about the potential pitfalls could help reduce postdischarge morbidity and overdose risk related to opioid use.

### Benefits of Patient Education for Postoperative Recovery

Multidisciplinary strategies that focus on opioid-sparing multimodal analgesic regimens, such as enhanced recovery after surgery programs, have been associated with decreased perioperative opioid consumption.^11^ There is also an increasing awareness of the beneficial role of preoperative education in improving various aspects of a patient’s perioperative journey.^12–14^ Preoperative educational interventions have been successfully implemented to help decrease postoperative pain,^29^ opioid use,^30^ and perioperative anxiety.^31^ The objective of this narrative review was to identify, examine, and summarize the available evidence on the use and effectiveness of preoperative educational interventions with respect to their impact on pain, opioid use, and psychological outcomes. The findings from this review will ultimately allow us to make more informed decisions regarding the most suitable types of preoperative educational tools and enable the creation of novel tools to improve patient recovery and functioning following surgery.

## Methods

Team members ran a MEDLINE ALL (Ovid Platform) database search from its inception (1946–May 24, 2022). The search strategy concept blocks were assembled on the topics of “preoperative” AND “patient education” AND “elective surgery,” limited to the English language, humans, and adults. Preliminary searches were performed and full-text literature was examined for keywords, controlled vocabularies, text word terms, and synonyms.

**Figure 1.**
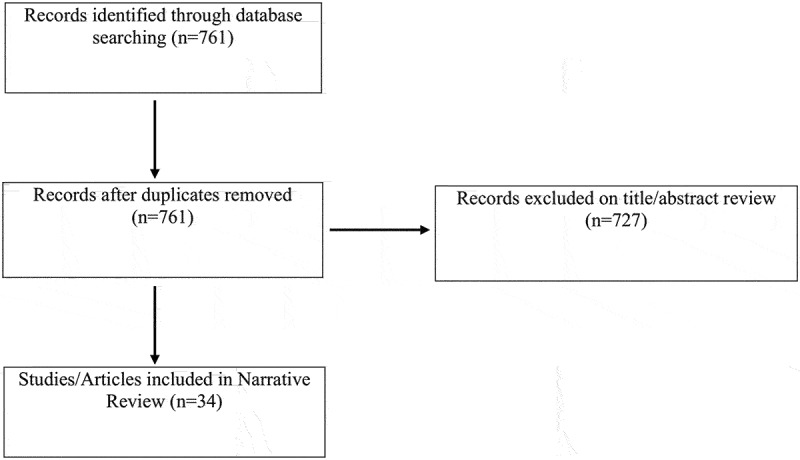
Flow diagram of included articles.

In total, the search yielded 761 citations after duplicates were accounted for (Figure 1). Titles and abstracts of identified studies were screened by two reviewers (H.Y. and P.J.). The full texts of eligible studies were retrieved and screened independently. If agreement could not be reached on whether a study should be included or excluded, a third reviewer (H.C.) reviewed the manuscript and made the final decision to include or exclude the study. Studies reporting on the impact of preoperative educational interventions on pain, and/or opioid use, and/or psychological outcomes were included. Studies were excluded if they reported on enhanced recovery after surgery protocols.

An extraction table was used to collect relevant data from each included study. Each entry included the title, type of study, sample size, intervention, and relevant findings. The results were subdivided into categories relevant to postsurgical outcomes of interest. The methodological quality of each study was also assessed. The Cochrane Risk of Bias v2 tool^32^ was used to assess randomized controlled trials and the Risk of Bias in Non-Randomized Studies of Interventions tool^33^ was used to assess nonrandomized prospective studies.

## Results

### Overview of Articles

Thirty-three of the included studies were randomized controlled trials and one was a prospective controlled trial. Twelve studies examined the impact of preoperative educational interventions on postoperative pain, 8 examined the impact of preoperative educational interventions on postoperative opioid use, and 24 studies evaluated postoperative psychological outcomes. Details of the included studies are summarized in Table 1.

**Table 1.** Summary of relevant details of each included study.

Author	Title	Type of Surgery	Study Design	Participants completed	Educational Intervention	Delivery Format	Last Post-op Measure Time Point	Relevant Findings	Outcomes: (+) positive, (=) no effect, or (-) negative
Ahmed et al.^64^	Effect of a patient-information video on the preoperative anxiety levels of cataract surgery patients	Ophthalmology	Prospective controlled trial	N=200	Format: video (length not specified)Topic: explanation of process of routine cataract surgery; individualpatients describing their cataract surgery experience	Video	day of surgery	Significant difference in anxiety scores between the control group and the intervention group	psychological (+)
Alter & Ilyas^50^	A Prospective Randomized Study Analyzing Preoperative Opioid Counseling in Pain Management After Carpal Tunnel Release Surgery.	Orthopedic	RCT	N=40	Format: opioid counseling by reviewing 1-page informational form with the surgeonTopic: explaining Opioid Epidemic, preoperative recommendations, risk factors for opioid abuse, nonopioid therapy options, duration of typical opioid consumption after surgery, lowest dose opioid prescribed, other opioid painkillers used	In-person; Paper	3 days	Patients in counseling group consumed significantly fewer opioid pills and total pain pills compared to control while experiencing no significant difference in pain levels	opioid use (+), pain (=)
Billquist et al.^48^	Pre-operative guided imagery in female pelvic medicine and reconstructive surgery: a randomized trial.	Gynecology	RCT	N=38	Format: 15 min audio recording (guided imagery) for 7 days prior to surgeryTopic: detailed day of surgery events and expectations using both guided imagery and relaxation techniques.	Audio	6 weeks	Guided imagery improved patient preparedness for pelvic floor surgery on day of surgery and 6 weeks post operatively	psychological (+)
Campagna et al.^57^	Impact of a Preoperative Video-Based Educational Intervention on Postoperative Outcomes in Elective Major Abdominal Surgery: a Randomized Controlled Trial	General Surgery	RCT	N=160	Format: video + in-person opportunity to ask questions about doubtsTopic: information about preoperative (e.g., ward structure, preparation for surgery), perioperative (e.g., operating theater setting, awakening), and postoperative period (e.g., devices used, potential complications); participants had opportunity to discuss any doubts that arose from video	Video; In-person	4 days	No differences between groups in postoperative anxiety or pain	psychological (=), pain (=)
Che et al.^38^	Effects of an Informational Video About Anesthesia on Pre- and Post-Elective Cesarean Section Anxiety and Recovery: A Randomized Controlled Trial	Obstetric	RCT	N=121	Format: 8.5 min videoTopic: process of administering anesthesia & equipment, nurses opening intravenous channels, anesthesiologists administering anesthesia, process of returning to the ward, responsibilities of anesthesiologist, preoperative and intraoperative rights of mothers, after-effects of anesthesia	Video	2 days	Watching a video about anesthesia significantly reduced anxiety levels of parturient women during the perioperative period	psychological (+)
Cheesman et al.^45^	The effect of preoperative education on opioid consumption in patients undergoing arthroscopic rotator cuff repair: a prospective, randomized clinical trial-2-year follow-up	Orthopedic	RCT	N=140	Format: 2 min video; paper outline of video key pointsTopic: recommended postoperative opioid use, side effects, dependence, and addiction	Video; Paper	2 years	Study patients had lower rate of opioid dependence than control patients; significantly fewer prescriptions were filled, fewer pills were consumed, and fewer morphine milligram equivalents were consumed by study patients than by controls	opioid use (+)
Eley et al.^42^	Effect of an anaesthesia information video on preoperative maternal anxiety and postoperative satisfaction in elective caesarean section: a prospective randomized trial.	Obstetric	RCT	N=110	Format: 4.5 min videoTopic: patient journey from time of administration of anesthetic to arrival in post-anaesthetic care unit; animations used to explain basic anatomy of neuraxial anaesthesia; explanations and demonstrations of anesthesia procedures; descriptions of common sensations experienced after neuraxial anaesthesia for caesarean section	Video	1 day	No difference in anxiety, maternal satisfaction score between intervention and control groups; maternal satisfaction with neuraxial blockade for elective caesarean is high and not improved by anaesthesiainformation video	psychological (=)
Fahimi et al.^34^	The Effects of Multimedia Education on Postoperative Delirium in Patients Undergoing Coronary Artery Bypass Graft: A Randomized Clinical Trial	Cardiac	RCT	N=110	Format: 3 short videos of 4-6 minTopic: information about surgical procedure and recovery	Video	4 days	Significantly lower incidence of post-operative delirium in the morning of the second, third and fourth days after surgery in patients who received education compared to control group.	psychological (+)
Hoorntje et al.^37^	Goal Attainment Scaling Rehabilitation Improves Satisfaction with Work Activities for Younger Working Patients After Knee Arthroplasty: Results from the Randomized Controlled ACTION Trial	Orthopedic	RCT	N=120	Format: extended “Goal attainment scaling” (GAS) prehab programTopic: patient and the physiotherapist formulated 3 postoperative activity goals; a multidisciplinaryteam assessed the goals forapplicability and feasibility; a postoperative rehabilitation scheme was designed	In-person	12 months	Patient satisfaction with work activities was significantly higher in the GAS group compared to controls; Patient satisfaction with activities of daily living and leisure-time activities did not differ between groups.	psychological (+)
Ilyas et al.^58^	The Effect of Preoperative Opioid Education on Opioid Consumption After Outpatient Orthopedic Surgery: A Prospective Randomized Trial	Orthopedic	RCT	N=237	Format: brief video (length not specified)Topic: Opioid Epidemic, risk factors for opioid abuse, safe opioid consumption, info on the specific opioid the patient was prescribed, use of nonopioid therapy, instructions to call if adverse event	Video	not specified	Education significantly reduced number of opioid pills and total morphine equivalents consumed compared to control, with no negative effects on pain experience	opioid use (+), pain (=)
Kalogianni et al.^53^	Can nurse-led preoperative education reduce anxiety and postoperative complications of patients undergoing cardiac surgery?	Cardiac	RCT	N=395	Format: nurse-led preoperative education, including paper booklet and in-person education (20 to 40 min)Topic: procedural, psychoeducational and skills education; nurses emphasised breathing exercises, rising from bed, leg exercises, pain management, coughing, control of anxiety and movement; nurses responded to patients’ questions.	In-person; Paper	at discharge	Intervention reduced anxiety and postoperative complications, but was not effective in reducing readmissions of length of stay	psychological (+)
Kiran et al.^62^	The role of Rajyoga meditation for modulation of anxiety and serum cortisol in patients undergoing coronary artery bypass surgery: A prospective randomized control study	Cardiac	RCT	N=150	Format: three basic lessons of Rajyoga meditation training provided (instructor not specified) Topic: 1) self-realization and self-confidence and self-improvement by positive and genuine thinking, 2) charging the self (mind and intellect) by Supreme power, 3) positive and purposeful attitude self-awareness and self-empowerment	In-person	5 days	Patients who underwent Rajyoga training had significantly lower anxiety in comparison to the control group; serum cortisol levels favorably modulated by the practice of Rajyoga meditation	psychological (+)
Klaiber et al.^41^	Impact of preoperative patient education on the prevention of postoperative complications after major visceral surgery: the cluster randomized controlled PEDUCAT trial	General Surgery	RCT (cluster)	N= 244	Format: 1 hr seminar delivered by nursing staffTopic: measures to prevent postoperative complications, principles of acute pain therapy and coping strategies; patients were introduced to breathing exercises, careful postoperative out-of-bed mobilization, and practical post-op exercises.	In-person	30 days	No statistically significantdifference between the two groups in anxiety; depression scores were significantly higher in control group than intervention group on POD 30; pain scores were comparable betweenthe two groups, but on POD 7, pain intensity wassignificantly higher in intervention group	psychological (=/+), pain (=/-)
Korkmaz et al.^44^	An Evaluation of the Influence of Web-Based Patient Education on the Anxiety and Life Quality of Patients Who Have Undergone Mammaplasty: a Randomized Controlled Study	General Surgery	RCT (3 groups)	N=75	Format: web application vs. brochureTopic: pre-operativepreparation, pre-operative exercises, post-operative considerations,post-operative exercises, postsurgery therapies, andbreast self-examination	Web App or Paper	1 month	Differences in state of anxiety scores 1 day before surgery, 2nd day after surgery, and 1 month after surgery were statistically lower in the web-based education group than in the other two groups; differences in trait anxiety scores after 1 month were lower in the web and brochure groups than the control group	psychological (+)
Lai et al.^35^	Effect of preoperative education and ICU tour on patient and family satisfaction and anxiety in the intensive care unit after elective cardiac surgery: A Randomized Control Trial	Cardiac	RCT	N=100	Format: 15 min video + ICU tour by nurse or physicianTopic: ICU environment, types of invasive tubes and lines, postoperative pain management, medical management, communication between patients, family members and ICU staff, family support	Video; In-person	7 days	Education significantly improved patient and family satisfaction levels and was associated with decreased anxiety levels but not depression levels among patients.	psychological (+)
Lee et al.^47^	Effects of Educational Intervention on State Anxiety and Pain in People Undergoing Spinal Surgery: A Randomized Controlled Trial	Neurosurgery	RCT	N=86	Format: in-depth booklet, videos, images and in-person content review with surgical nurseTopic: introduction to disease, OR environment, surgical procedure, post-op care	Paper; In-person; Video	1 day	Pain and anxiety scores were lower in intervention group compared to controls	pain (+), psychological (+)
Lin et al.^54^	The effect of an anaesthetic patient information video on perioperative anxiety: A randomized study	Combined	RCT	N=100	Format: 8 min videoTopic: assessment at preoperative clinic and preoperative care, conduct of general anaesthesia, conduct of spinal anaesthesia, administration of sedation; postoperative care, including the management of pain and postoperative nausea and vomiting	Video	3 days	State anxiety was lower in intervention group than in control group just before surgery and the day after surgery; overall satisfaction was significantly higher in intervention group than control	psychological (+)
Mousavi Malek et al.^61^	Effects of Nurse-Led Intervention on Patients' Anxiety and Sleep Before Coronary Artery Bypass Grafting	Cardiac	RCT	N=160	Format: two 45-60 min education & support sessions delivered by nurseTopic: explanation of procedure, support patient's anxiety, fear, and its causes and correcting patients’ misconceptions; introduction & practice of stress management and relaxation methods	In-person	day of surgery	Significantly decreased anxiety and better quality of sleep in intervention group compared to control	psychological (+)
Palmer et al.^63^	Effects of Music Therapy on Anesthesia Requirements and Anxiety in Women Undergoing Ambulatory Breast Surgery for Cancer Diagnosis and Treatment: A Randomized Controlled Trial	General Surgery	RCT (3 groups)	N=207	Format: 5 min music (live or recorded) + conversation with music therapistTopic: discussion of choice of preferred music & integrated processing of selected song	Audio; In-person	at discharge	Compared with the control group, both patient-selected live music (LM) group and patient-selected recorded music (RM) groups had greater reductions in anxiety scores preoperatively; LM and RM groups did not differ from control group in recovery time; LM group had a shorter recovery time compared with the RM group; satisfaction scores for the LM and RM groups did not differ from those of control	psychological (+)
Paskey et al.^59^	Prospective Randomized Study Evaluating the Effects of Preoperative Opioid Counseling on Postoperative Opioid Use after Outpatient Lower Extremity Orthopedic Surgery	Orthopedic	RCT	N=107	Format: 5 min videoTopic: nature of opioids, Opioid Epidemic, safe opioid consumption, nonopioid pain management, avoiding opioid dependence	Video	14 days	Preoperative opioid counselling resulted in significant reduction in postoperative opioid consumption compared to control	opioid use (+)
Peng et al.^60^	Preoperative communication with anesthetists via anesthesia service platform (ASP) helps alleviate patients' preoperative anxiety	General Surgery	RCT	N=222	Format: anesthesia service platform web applicationTopic: customized info accessible via social media and curated by anesthetist; information about anesthesia, preoperative preparation, anesthetics or anesthetic manipulations, postoperative pain management	Web App	12 months	Patients in the intervention group had significantly lower anxiety levels, shorter length of stay, and higher well-being scores than controls, consumed fewer analgesics, but suffered more pain	psychological (+), pain (-)
Pereira et al.^52^	Preoperative anxiety in ambulatory surgery: The impact of an empathic patient-centered approach on psychological and clinical outcomes	General Surgery	RCT	N=104	Format: nurse-led empathic patient-centered educationTopic: eliciting and exploring patients’ questions and personalconcerns about surgery, addressing questions and concerns in a customized fashion through delivery of personalized information and empathic response to emotions through explicit appreciation of these emotions, leading to a sense of validation and understanding	In-person	1 month	Intervention group showed lower levels of preoperativeanxiety, pain, better surgery recovery, higher levels of dailyactivity, satisfaction with the information received, and better would healing compared to controls	psychological (+), pain (+)
Platto et al.^46^	Animated video consultation for reducing pre-operative anxiety in dermatologic surgery	Dermatology	RCT (cross-over)	N=45	Format: 2 min videoTopic: Anesthesia, excision, repair, post-op wound care, pain management	Video	day of surgery	Anxiety scores in video group were lower than standard care group (not significantly different); after crossing over, the standard care group that received video intervention had significant improvement in anxiety	psychological (+)
Rajput et al.^31^	Effect of preoperative multimedia based video information on perioperative anxiety and hemodynamic stability in patients undergoing surgery under spinal anesthesia	Combined	RCT	N=80	Format: 12 min videoTopic: general information on spinal anesthesia, explanation of common myths of surgery and anesthesia	Video	1 day	Significantly less anxiety and hemodynamic instability in intervention group compared to controls	psychological (+)
Sawhney et al.^29^	A Pain Education Intervention for Patients Undergoing Ambulatory Inguinal Hernia Repair: A Randomized Controlled Trial	General Surgery	RCT	N=82	Format: booklet + 20 min individualized face-to-face education session with a nurse practitioner; two telephone support callsTopic: definition of pain, importance of managing pain after surgery, how to communicate pain intensity, commonly used analgesics, when to use analgesics, manage adverse effects of analgesics, non-pharmacological pain management; patient concerns regarding pain management	In-person; Paper; Phone	7 days	Improved pain scores on POD 2 in intervention group compared to control	pain (+)
Schmidt et al.^55^	Patient Empowerment Improved Perioperative Quality of Care in Cancer Patients Aged >= 65 Years - A Randomized Controlled Trial	Combined	RCT	N=105	Format: information booklet and diary keeping; encouragement by staff to ask questions and take an active role in rehabilitation Topic: information about surgery, anesthesia and perioperative management, acute postoperative pain therapy, mobilization, nutrition, and rehabilitation; life-style-related risks following discharge from the hospital described; information regarding, support groups and home care institutions offering specific assistance after discharge	Paper	12 months	Patients in the intervention group reported significantly less postoperative pain than the control group	pain (+)
Stepan et al.^36^	Standardized Perioperative Patient Education Decreases Opioid Use after Hand Surgery: A Randomized Control Trial	Orthopedic	RCT	N=191	Format: 7 min min video + laminated postoperative pain cardTopic: postoperative pain management, expected pain after surgery, Opioid Epidemic, side effects of opioids, tiered system of pain management	Video; Paper	15 days	Education group more likely to take no opioid medication and took significantly fewer opioid pills than control group	opioid use (+)
Strøm et al.^40^	A web-based platform to accommodate symptoms of anxiety and depression by featuring social interaction and animated information in patients undergoing lumbar spine fusion: a randomized clinical trial	Neurosurgery	RCT	N=114	Format: web-based interactive platformTopic: information aimed to reduce anxiety, catastrophic thoughts, surgery misconceptions; explanation of course of treatment & rehab	Web App	6 months	Providing patients with access to web platform had no effect on the hospital anxiety and depression scale scores at 2 days, 3 months or 6 months after surgery; no significant differences in pain between groups at 3 or 6 months after surgery, but the intervention group had lower pain scores at 2 days	psychological (=) pain (=/+)
Sugai et al.^56^	The importance of communication in the management of postoperative pain	Combined	RCT	N=135	Format: two 15-30 min education sessions led by a surgeon involving in-person instruction and handoutTopic: how body responds to pain, and how endorphins cause natural analgesia; negative effects that narcotics have on endorphin production and activity, mechanisms of non-opioid analgesics	In-person; Paper	5 days	90% of patients in intervention group declined a prescription for hydrocodone whereas 100% of controls filled their hydrocodone prescriptions; intervention group had significantly lower pain scores and shorter duration of pain than control	opioid use (+), pain (+)
van Eck et al.^31^	Web-Based Education Prior to Outpatient Orthopaedic Surgery Enhances Early Patient Satisfaction Scores: A Prospective Randomized Controlled Study	Orthopedic	RCT	N=177	Format: interactive web-based toolTopic: preoperative instructions, day-of-surgery expectations, postoperative instructions (including tips for pain control); links to videos demonstrating an example of the surgery that the patient is to undergo	Web App	2 weeks	Patient satisfaction score was significantly higher in the intervention group compared to control group	psychological (+)
Vincent et al.^43^	Prospective Randomized Study Examining Preoperative Opioid Counseling on Postoperative opioid Consumption after Upper Extremity Surgery	Orthopedic	RCT	N=131	Format: 5 min videoTopic: nature of opioids, Opioid Epidemic, safe opioid consumption, nonopioid pain management, avoiding opioid dependence	Video	14 days	Education group consumed significantly fewer opioids compared to controls; no difference in pain scores between groups	opioid use (+),pain (=)
Wongkietkachorn et al.^49^	Preoperative Needs-Based Education to Reduce Anxiety, Increase Satisfaction, and Decrease Time Spent in Day Surgery: A Randomized Controlled Trial	Combined	RCT	N=450	Format: verbal & written information based on patient needs delivered by physicianTopic: disease info, procedural detail, complications, patient behavior, pain	In-person; Paper	after surgery (not specified)	Significantly greater decrease in anxiety and increase in satisfaction in the needs-based education compared to traditional education group; time spent on education in needs-based group significantly reduced compared to traditional	psychological (+)
Yuzkat et al.^39^	Effects of showing the operating room on preoperative anxiety and hemodynamics among patients with hypertension: A randomized controlled trial	Combined	RCT	N=90	Format: pre-op visit to OR	In-person	day of surgery	Showing the OR decreased preoperative anxiety, blood pressure, and heart rate; intervention also reduced number of postponed operations and increased patient satisfaction	psychological (+)
Zohar-Bondar et al.^30^	The Effect of Standardized perioperative patient Education on Opioid Use After Minor Soft Tissue Procedures Distal to the Wrist	Orthopedic	RCT	N=174	Format: 10 min video webinar + laminated postoperative pain cardTopic: expected pain after surgery, Opioid Epidemic, side effects of opioids, tiered system of pain management	Video; Paper	15 days	Education group took significantly fewer opioid pills than those in the standard care group	opioid use (+)

RCT: randomized control trial; OR: operating room; ICU: intensive care unit; POD: post-operative day

### Methodological Quality

The risk of bias assessment for the included studies is summarized in Tables 2 and 3. Of the randomized controlled trials, 11 of the studies were at low risk of bias,^29,30,34–42^ 17 studies had some concerns,^31,43–57^ and 6 studies were at high risk of bias.^58–63^ The one nonrandomized prospective study had a moderate risk of bias.^64^ The most common area of concern in study design was due to bias arising from the randomization process. Potential for bias in selection of the reported result was also common because many studies did not report information regarding preregistration of the study protocol.

**Table 2.** Risk of bias assessment using the Cochrane Risk of Bias v2 tool for randomized trials.

Study	Bias arising from the randomization process	Bias due to deviations from intended interventions	Bias due to missing outcome data	Bias in measurement of the outcome	Bias in selection of the reported result	Bias arising from the timing of recruitment of participants in a cluster-randomized trail	Bias arising from period and carryover effects	Overall risk of bias
Alter and Ilyas^50^	Some concerns	Low	Low	Low	Some concerns			Some concerns
Billquist et al.^48^	Low	Low	Low	Low	Some concern			Some concerns
Campagna et al.^57^	Some concerns	Low	Low	Some concerns	Some concerns			Some concerns
Che et al.^38^	Low	Low	Low	Low	Low			Low
Cheesman et al.^45^	Some concerns	Low	Low	Low	Some concerns			Some concerns
Eley et al.^42^	Low	Low	Low	Low	Low			Low
Fahimi et al.^34^	Low	Low	Low	Low	Low			Low
Hoorntje et al.^37^	Low	Low	Low	Low	Low			Low
Ilyas et al.^58^	High	Low	Low	Low	Some concerns			High
Kalogianni et al.^53^	Some concerns	Some concerns	Low	Low	Some concerns			Some concerns
Kiran et al.^62^	Low	Some concerns	Low	High	Some concerns			High
Klaiber et al.^41^	Low	Low	Low	Low	Low	Low		Low
Korkmaz et al.^44^	Some concerns	Low	Low	Low	Some concerns			Some concerns
Lai et al.^35^	Low	Low	Low	Low	Low			Low
Lee et al.^47^	Some concern	Low	Low	Low	Some concern			Some concerns
Lin et al.^54^	Some concerns	Low	Low	Low	Some concerns			Some concerns
Mousavi Malek et al.^61^	High	Low	High	Low	Low			High
Palmer et al.^63^	Low	Low	Low	High	Some concerns			High
Paskey et al.^59^	High	Low	Low	Low	Low			High
Peng et al.^60^	Some concerns	Low	Low	High	Low			High
Pereira et al.^52^	Some concerns	Some concerns	Low	Low	Some concerns			Some concerns
Platto et al.^46^	Some concerns	Some concerns	Low	Low	Some concerns		Low	Some concerns
Rajput et al.^31^	Low	Low	Low	Low	Some concerns			Some concerns
Sawhney et al.^29^	Low	Low	Low	Low	Low			Low
Schmidt et al.^55^	Some concerns	Low	Low	Low	Low			Some concerns
Stepan et al.^36^	Low	Low	Low	Low	Low			Low
Strøm et al.^40^	Low	Low	Low	Low	Low			Low
Sugai et al.^56^	Some concerns	Some concerns	Low	Low	Some concerns			Some concerns
van Eck et al.^31^	Some concerns	Low	Low	Low	Some concerns			Some concerns
Vincent et al.^43^	Some concerns	Low	Low	Some concerns	Some concerns			Some concerns
Wongkietkachorn et al.^49^	Some concerns	Low	Low	Low	Low			Some concerns
Yuzkat et al.^39^	Low	Low	Low	Low	Low			Low
Zohar-Bondar et al.^30^	Low	Low	Low	Low	Low			Low

**Table 3.** Risk of bias assessment using the Risk of Bias in Non-randomized Studies of Interventions tool.

Study	Bias due to confounding	Bias in selection of participants into the study	Bias in classification of interventions	Bias due to deviations from intended interventions	Bias due to missing data	Bias in measurement of outcome	Bias in selection of reported result	Overall risk of bias
Ahmed et al.^64^	Moderate	Moderate	Low	Low	Low	Low	Low	Moderate

### Context

A large proportion of included studies were conducted within the United States,^30,36,43,45,46,48,50,51,56,58,59,63^ with other studies originating in Iran,^34,61^ India,^31,62^ China,^38,60^ Turkey,^39,44^ the United Kingdom,^64^ Germany,^41,55^ Taiwan,^47,54^ Hong Kong,^35^ the Netherlands,^37^ Denmark,^40^ Canada,^29^ Portugal,^52^ Thailand,^49^ Greece,^53^ Australia,^42^ and Italy.^57^ Sample sizes ranged from 38 to 652 participants, aged 18 years and older, with an equal proportion of men and women, except for obstetric studies.

### Preoperative Interventions

Multimedia resources were the most common format for delivering preoperative education among the included studies. Informational videos were most frequently used, with ten studies that used videos only,^31,34,38,42,43,46,54,58,59,64^ three that combined videos and paper resources,^30,36,45^ two that combined video with in-person education,^35,57^ and one that used video, paper, and in-person education.^47^ Four studies^40,44,51,60^ delivered educational interventions using interactive web applications, three of which included videos^44,51,60^ and one that featured a social media interface for interactions with health care providers.^60^ Two studies used audio-based resources.^48,63^

Six studies implemented in-person education only^37,39,41,52,61,62^ and another five added paper resources to in-person education,^29,49,50,53,56^ including brochures, booklets, and information cards, with one of these studies also including follow-up phone calls.^29^ Only one study relied solely on a paper resource.^55^ In-person education was typically delivered by a healthcare provider, including nursing staff,^29,35,41,47,52,53,61^ physicians (e.g., anesthesiologists, surgeons),^35,39,49,50,56^ a physiotherapist,^37^ and a music therapist.^63^ Two studies used a patient-centered technique,^49,52^ providing patients with the information necessary to participate in medical decision making. In one study the interventional group had a tour of the operating theater prior to surgery.^39^ Two studies used comfort therapy as an intervention, including one that used meditation^62^ and another that used music therapy.^63^ One study used hypnosis in the form of guided imagery,^48^ one used psychosocial therapy/counseling,^50^ and another used goal attainment and physiotherapy^37^ as interventions.

Delivery and timing of preoperative interventions varied among studies, with some taking place on the day of surgery and others delivered days in advance. Duration of interventions ranged between 2 min and 60 min. All studies implemented interventions preoperatively, except for Palmer et al.,^63^ in which music therapy was implemented pre- and intraoperatively.

Studies that included data on postoperative opioid consumption used surveys and logs to track patient medication consumption information. Validated questionnaires used included a numerical rating scale, a visual analogue scale, and the Brief Pain Inventory, all of which were predominantly used in studies that focused on postoperative pain. Postoperative cognitive and psychological outcomes were assessed by the following scales: Confusion Assessment Methods for the Intensive Care Unit, Patient Satisfaction in the Intensive Care Unit, Family Satisfaction in the Intensive Care Unit, Amsterdam Preoperative Anxiety and Information Scale, State-Trait Anxiety Inventory, Hospital Anxiety and Depression Scale, Short Form Survey, Groningen’s Sleep Quality Scale, and the Outpatient and Ambulatory Surgery Consumer Assessment of Healthcare Providers and Systems survey.

## Outcomes

### Postoperative Pain

Twelve studies reported information on postoperative pain.^29,40,41,43,47,50,52,55–58,60^ Five of these studies^29,47,50,52,55,56^ found a significant reduction in postoperative pain after preoperative education, and all involved in-person interaction with a health care provider. Sawhney et al.^29^ evaluated the effectiveness of an individualized hernia repair education intervention for patients undergoing inguinal hernia repair. The hernia repair education intervention included written and verbal information delivered by a nurse regarding managing pain as well as two telephone support calls. At day 2, the intervention group reported significantly lower scores across pain intensity outcomes, including worst 24-h pain on movement and at rest, and pain now on movement and at rest, in comparison to the control group. Pereira et al.^52^ also examined the benefits of nurse-led empathic patient-centered education in patients undergoing ambulatory surgery. They found that the intervention group had lower pain levels on the second postoperative day compared to the control group.

In a study by Sugai et al.,^56^ a surgeon delivered in-person education and reviewed a written handout with patients 2 weeks before outpatient surgery. The topic of education consisted of information about how the body responds to pain and how endorphins cause natural analgesia. The intervention group had significantly lower average pain scores than the control group and a shorter duration of pain. Lee et al.^47^ employed an educational intervention in patients undergoing spinal surgery involving an in-depth booklet and videos on topics related to the disease, surgery, and postoperative care. A surgeon also reviewed the content in person with patients. The study found that pain levels were significantly lower for the interventional arm of the study compared to standard care.

Schmidt et al.^55^ also delivered preoperative education using a patient empowerment information booklet with in-depth information regarding surgery, anesthesia, pain management, and rehabilitation. Patients were aged 65 and older and undergoing elective surgery for gastrointestinal, genitourinary, and thoracic cancer. In addition to receiving the booklet, patients were also asked to keep a diary and repeatedly encouraged to consult with the health care team regarding medication and rehabilitation. Patients in the intervention group reported significantly less postoperative pain than the control group that received standard care.

Four studies found no effect of preoperative education on postoperative pain,^43,50,57,58^ and one study reported mixed results.^40^ Three of the studies that found no effect used videos as the educational intervention.^43,57,58^ Vincent et al.^43^ employed a 5-min video to deliver information about safe opioid use and nonopioid pain management to patients undergoing upper extremity surgery. They found no differences in pain scores between the education and the control groups. Similarly, Ilyas et al.^58^ used a brief video to deliver education on the same topics to patients undergoing outpatient orthopedic surgery. They also found no significant effects on the experience of pain between the intervention and control groups. Campagna et al.^57^ also employed a video of unspecified length to deliver information regarding the perioperative experience. In addition, they gave patients an opportunity to ask questions about any doubts they had regarding the content of the video. They reported that postoperative pain was well controlled among female surgical patients presenting for colorectal surgery secondary to gastrointestinal cancer and that there were no differences found in postoperative pain in the education group compared to the control group.

A study by Alter and Ilyas^50^ did not use a video but instead examined the benefits of surgeon-delivered education alongside a paper handout on the topic of opioid use and pain management in patients undergoing carpal tunnel release surgery. They found no significant differences in pain levels between the intervention and control groups. Strøm et al.^40^ employed an interactive web platform to deliver education and found no improvement in pain at 3 months and 6 months after lumbar spine fusion surgery, but they did observe improved pain at 2 days after surgery.

Importantly, three of the above studies that found no effect on pain also examined opioid use following the educational intervention, and all found a decrease in postoperative opioid consumption (see below in Postoperative Opioid Use).^43,50,58^ These findings demonstrate that pain levels were not affected despite patients using less opioid medication postoperatively.

Two studies found an increase in reported postoperative pain following preoperative educational interventions. Peng et al.^60^ found that preoperative education using an anesthesia service platform was effective in preventing anxiety in female patients before laparoscopic cholecystectomy, including improving patients’ general well-being and shortening their length of stay, but patients undergoing the intervention reported higher postoperative pain levels. Klaiber et al.^41^ found mixed results following a 1-h nurse-led seminar on preventing postoperative complications and coping strategies for pain. In a cluster-randomized trial, patients undergoing major visceral surgery who received education had comparable pain scores to control patients on postoperative days 2 and 7, except patients in the education group reported higher scores with respect to pain intensity on day 7. The above studies highlight that some negative effects with respect to the experience of postoperative pain following educational interventions can occur.

### Postoperative Opioid Use

A total of eight studies reported on postoperative opioid consumption.^30,36,43,45,50,56,58,59^ All of these found that perioperative patient education significantly reduced postoperative opioid use.

Six studies employed videos about safe opioid use as the educational intervention.^30,36,43,45,58,59^ In a study by Vincent et al.^43^ of patients undergoing outpatient upper extremity surgery, patients who received preoperative opioid counseling in the form of a 5-min video consumed significantly fewer opioids postoperatively, 93.7 morphine equivalent units compared to 143.4 morphine equivalent units in the control group. There was no difference in pain at any point between groups. In a study by Stepan et al.,^36^ all patients scheduled to undergo ambulatory hand surgery received a webinar video with instructions for study participation, and the education group received an additional 10 min of instruction on postoperative pain management plus a postoperative pain management reference card. Patients in the education group were more likely to take no opioid medication (42% versus 25%) and took significantly fewer opioid pills than those in the control group. Zohar-Bondar et al.^30^ randomized patients scheduled to undergo elective outpatient surgery, comprising soft tissue procedures distal to the wrist, to either receive pain management education or standard of care. As in Stepan et al.,^36^ all patients viewed a webinar video before surgery, with the education group having an additional 10 min of education and receiving a pain management reference card for review after surgery. Patients in the education group took significantly fewer opioid pills (median = 0, range 0–13) than those in the standard group (median = 0.5, range 0–40), although opioid consumption was low in both groups likely due to the minor nature of the surgical procedure.

In a study by Paskey et al.,^59^ preoperative opioid counseling was delivered via a 5-min video. Patients undergoing elective outpatient lower extremity orthopedic surgery who received the preoperative opioid counseling consumed on average 6.5 opioid pills in comparison to the control group, which consumed on average 12.4 pills. In a study by Ilyas et al.,^58^ patients undergoing outpatient orthopedic surgeries who also received preoperative opioid education in the form of a brief video consumed significantly fewer opioids (6 pills) when compared with the group not receiving education (12 pills). This finding was consistent across both upper and lower extremity surgery.

A study by Cheesman et al.^45^ examined the long-term effects of preoperative opioid education delivered in the form of a 2-min video and a paper outline of the key points in the video. They examined postoperative opioid consumption after arthroscopic rotator cuff repair at 2-year follow-up. They found that preoperative opioid education had significant benefits for patients in the education cohort compared to the control cohort, including a lower rate of opioid dependence, fewer filled prescriptions for opioids, and lower consumption of opioids.

In two studies, preoperative opioid education was delivered in person by a surgeon.^50,56^ A study by Alter and Ilyas provided in-person counseling and a one-page information form regarding opioid use and nonopioid therapy options.^35^ They found that patients in the counseling group consumed significantly fewer opioid pills and fewer total pain pills compared to control patients, with no significant difference in reported pain levels. Similarly, in a study by Sugai et al.,^56^ participants in the experimental group received in-person and written forms of patient education 2 weeks before outpatient surgery consisting of information about how the body responds to pain and how endorphins cause natural analgesia. Ninety percent of subjects in the experimental group declined a prescription for hydrocodone, whereas 100% of participants in the control group filled their hydrocodone prescriptions. However, the study did not specify whether the control group was given the option to decline the hydrocodone prescription in the same way that the experimental group was. Importantly, the study also did not provide information on how much hydrocodone was consumed by control participants, and only 20% requested a refill of hydrocodone. There was also little information provided regarding the types of surgeries performed and how they were distributed between the two groups. The control group was also younger than the experimental group, which may have confounded the results because younger age is a known risk factor for opioid use after surgery.^65^

### Psychological Outcomes

Twenty-four studies^31,34,35,37–42,44,46–49,51–54,57,60–64^ reported data on postoperative psychological outcomes, and 20 of these found positive effects of preoperative educational interventions.^31,34,35,37–39,44,46–49,51–54,60–64^ Fourteen of these studies found a significant decrease in postoperative anxiety in participants exposed to perioperative education.^31,35,38,39,44,46,52–54,60–64^ For instance, Pereria et al.^52^ found that an empathic patient-centered intervention reduced anxiety and increased surgical recovery scores, wound healing, and patient satisfaction. They postulated that improved surgical recovery and wound healing could be a direct result of the empathic patient-centered approach or could be mediated by decreased preoperative anxiety.

Postoperative patient satisfaction scores were reported in nine studies,^35,37,39,42,49,51,52,54,63^ and seven of these studies^35,37,39,49,51,52,54^ reported improved scores after receiving preoperative education, whereas the remaining two studies^42,63^ saw no effect.

Fahimi et al.^34^ observed a reduction in postoperative delirium following a preoperative educational intervention to help patients undergoing coronary artery bypass surgery familiarize themselves with the surgical and intensive care unit (ICU) environment and procedures. The authors postulated that increased familiarity with the ICU resulting from the preoperative intervention contributed to a lower incidence of delirium on days 2 to 4 after surgery. Billquist et al.^48^ found improved patient preparedness after preoperative education.

In three studies,^40,42,57^ no differences were found in postoperative psychological outcomes after preoperative education. Strøm et al.^40^ examined the effect of a web-based Spine Platform featuring Interaction and Information by Animation on symptoms of anxiety, depression, pain, disability, and health-related quality of life. They found no statistically significant difference between the web-based Spine Platform featuring Interaction and Information by Animation group and the control group regarding Hospital Anxiety and Depression Scale scores at 3-month follow-up. Similarly, Eley et al.^42^ and Campagna et al.^57^ found no effect of education on postoperative anxiety. Eley et al. also saw no improvement in satisfaction following preoperative education for women undergoing elective cesarean section and observed that satisfaction was already high in this study population.

Klaiber et al.^41^ found mixed effects for psychological outcomes. Anxiety levels on day 7 and day 30 after surgery did not differ in patients undergoing major visceral surgery, whether they were provided with preoperative education or not. However, they did observe lower depression scores on day 30 in the group that received education compared to standard care.

## Discussion

Overall, this narrative review found that preoperative education interventions are beneficial in reducing postsurgical opioid consumption and improving psychological outcomes, especially anxiety. There is also evidence of benefits for postoperative pain, but studies are more mixed in this regard. Preoperative educational interventions are effective strategies that can be used to enhance patient safety and overall psychological well-being.

Five of 12 studies that examined effects on pain reported a reduction in postoperative pain after integrating perioperative education,^29,47,50,52,55,56^ while four studies found no effect on pain,^43,50,57,58^ one study found mixed effects,^40^ and two studies found evidence for a negative effect.^41,60^ Studies that showed a benefit of educational interventions for postoperative pain relied primarily on in-person delivery of education, as opposed to video-based interventions or web applications primarily used in the other studies. Controlled studies that more carefully examine how the format of preoperative education influences postoperative pain are therefore warranted to find the best protocol for improving this outcome.

All studies that evaluated postoperative opioid consumption found that perioperative education significantly reduced postoperative opioid use without adverse effects on postoperative pain.^30,36,43,45,50,56,58,59^ All of these studies focused the topic of education on safe postoperative opioid use and nonopioid approaches to pain management, and most relied on brief videos to deliver the educational intervention. These findings demonstrate that preoperative opioid education is feasible and effective in reducing subsequent opioid use, providing a promising avenue toward minimizing postoperative opioid consumption.

Psychological outcomes were by far the most common outcome reported (i.e., 24 articles), with significant improvements noted with respect to anxiety^31,35,38,39,44,46,52–54,60–64^ and patient satisfaction^35,37,39,49,51,52,54^ across many of the studies. There was no one approach to the format and topic of education that stood out at as superior in this domain, suggesting that there is flexibility in selecting the appropriate educational approach to implement preoperatively based on the available resources and constraints of the surgical service.

Most of the studies included in this review relied on short educational interventions that were practical to implement as part of the process of preparing patients for surgery. There was some evidence that in-person interventions delivered by a health care provider were more effective for improving postoperative pain, whereas using a brief educational video was sufficient to have a positive impact on reducing postoperative opioid consumption. Out of 34 studies, only 2 showed no effect of the education intervention in any domain. These findings suggest that implementing a preoperative educational intervention as part of routine preoperative care is feasible and has a high probability of success.

## Limitations

Although MEDLINE ALL is a very robust database, we may not have identified all articles available on the topic by searching only this database. This narrative review was limited to studies that met the current inclusion criteria.

Among the included studies, the length of follow-up was variable, and there were limited data regarding long-term benefit. Only four studies measured outcomes beyond the first 3 months after surgery,^37,40,45,55^ with two studies finding that preoperative educational interventions had long-term benefits at 12 months^37^ and up to 2 years^45^ and the other two finding no differences in outcomes at 6 months^40^ and 12 months.^55^ Orthopedics was the most common surgical specialty represented among the included studies, which may limit the overall applicability of the results. In some studies, clinical details, such as patient demographic characteristics, history of chronic pain, and type of opioid prescribed were not available.

There were also concerns regarding the methodological quality of two-thirds of the included studies. Many studies provided limited details regarding the randomization and concealment process or used approaches that did not reflect assignment at random (e.g., assignment by year of birth). Proper blinding of participants and health care providers was also a notable challenge with study design across many of the include studies. Participants and health care providers were often aware of the participants’ assigned condition by virtue of implementing the educational intervention. Only a few studies took care to design an intervention that appeared similar in both the experimental and control groups. For instance, Stepan et al.^36^ and Zohar-Bondar et al.^30^ used a video intervention where both groups watched a video about study participation but the intervention group also received education as part of the video content. This approach allowed for more careful assessment of the influence of educational content itself and proper blinding.

Overall, more rigorous methodological approaches are required in future research in this area.

## Future Directions

Opioids have played an integral role in treating acute postoperative pain, and surgery remains one of the most common indications for the initiation of opioids.^1^ Prescribing habits vary worldwide, with North American surgeons out-prescribing other parts of the world,^66^ and Canada ranking second in the number of opioids prescriptions per capita.^21^ Thus, opioid prescribing has remained a prominent public health concern and a contributing factor to the ongoing opioid epidemic. Perioperatively, physicians have responded to this crisis by creating new opioid prescribing guidelines.^24^ Organizations, including the Centers for Disease Control and Prevention, have continued to support health care systems with data, tools, and guidance for evidence-based decision making with aims to improve opioid prescribing and patient safety. A part of this initiative includes increasing public awareness about prescription opioids to encourage safe choices regarding opioid consumption.^67^ The preoperative period is a pivotal time point to initiate patient education and promote greater awareness of opioid safety.

As we continue to perform millions of major surgical interventions annually, it behooves perioperative health care practitioners to create perioperative education resources. The cost to society for poor surgical outcomes is estimated to be billions of dollars per year.^68^ Every attempt should be made to create low-cost solutions to such a major health care challenge. There is currently considerable interest in building transitional pain services that are focused on identifying and treating patients at risk of developing chronic postsurgical pain and persistent opioid use.^9^ The results of this review suggest that presurgical educational tools could be a useful addition to these types of services and provide value for patients set to undergo a surgical intervention. A strategy that provides essential information to patients about their surgical journey, including postoperative pain and safe opioid use, and helps to allay patients’ anxiety and fears prior to surgery would pay dividends going forward. Ultimately, a nationally available preoperative educational resource would have a beneficial impact on reducing pain-related disability and persistent opioid use in surgical patients across Canada.

The multistakeholder national initiative, Pain Canada, supported by Pain BC, aims to develop preoperative education modules that can be accessible to all Canadians scheduled to have surgery. We hope that these online modules will help to prepare patients psychologically and provide them with information about what to expect during the preoperative and postoperative periods. Delivering this type of educational content will aim to foster clearer expectations regarding the postsurgical recovery process and safe medication use.

## Conclusion

It is imperative that strategies continue to be implemented to reduce persistent opioid use following surgery, which could evolve into an opioid use disorder for some patients. Preoperative educational programs continue to show promise as an effective strategy aimed at protecting patients and improving healthcare in Canada. Though there are no concrete protocols that can be outlined from this narrative review, it is apparent that preoperative education can be consistently successful in helping to reduce opioid use and improve psychological outcomes, as well as having potential for improving postoperative pain. Future research is needed, aligned with novel educational content delivered in a timely manner in the preoperative setting. Based on this review, we would anticipate that these educational interventions will improve outcomes, positively impact patient psychological outcomes, reduce health care costs, and ultimately save lives.

## References

[1] Ladha KS, Neuman MD, Broms G, Bethell J, Bateman BT, Wijeysundera DN, Bell M, Hallqvist L, Svensson T, Newcomb CW, et al. Opioid prescribing after surgery in the United States, Canada, and Sweden. JAMA Network Open. 2019;2(9):e1910734. doi:10.1001/jamanetworkopen.2019.10734. 31483475 PMC6727684

[2] Katz J, Seltzer Z. Transition from acute to chronic postsurgical pain: risk factors and protective factors. Expert Rev Neurother. 2009;9(5):723–23. doi:10.1586/ern.09.20. 19402781

[3] Fletcher D, Stamer UM, Pogatzki-Zahn E, Zaslansky R, Tanase NV, Perruchoud C, Kranke P, Komann M, Lehman T, Meer W, et al. Chronic postsurgical pain in Europe: an observational study. Eur J Anaesthesiology. 2015;32(10):725–34. doi:10.1097/EJA.0000000000000319. 26241763

[4] Schug SA, Lavand’homme P, Barke A, Korwisi B, Rief W, Treede R-D. The IASP classification of chronic pain for ICD-11: chronic postsurgical or posttraumatic pain. PAIN. 2019;160(1):45–52. doi:10.1097/j.pain.0000000000001413. 30586070

[5] Rosenberger DC, Pogatzki-Zahn EM. Chronic post-surgical pain – update on incidence, risk factors and preventive treatment options. BJA Educ. 2022;22(5):190–96. doi:10.1016/j.bjae.2021.11.008. 35496645 PMC9039436

[6] Katz J, Weinrib AZ, Clarke H. Chronic postsurgical pain: from risk factor identification to multidisciplinary management at the Toronto General Hospital transitional pain service. Can J Pain. 2019;3(2):49–58. doi:10.1080/24740527.2019.1574537. PMC8730596 35005419

[7] Hah JM, Bateman BT, Ratliff J, Curtin C, Sun E. Chronic opioid use after surgery: implications for perioperative management in the face of the opioid epidemic. Anesth Analg. 2017;125(5):1733–40. doi:10.1213/ANE.0000000000002458. 29049117 PMC6119469

[8] Wylie JA, Kong L, Barth RJJ. Opioid dependence and overdose after surgery: rate, risk factors, and reasons. Ann Surg. 2022;276(3):e192. doi:10.1097/SLA.0000000000005546. 35837951

[9] Katz J, Weinrib A, Fashler SR, Katznelson R, Shah B, Ladak S, Jiang J, Li Q, McMillan K, Santa Mina D, et al. The Toronto General Hospital transitional pain service: development and implementation of a multidisciplinary program to prevent chronic postsurgical pain. J Pain Res. 2015;8:695–702. doi:10.2147/JPR.S91924. 26508886 PMC4610888

[10] Shipton EA. The transition of acute postoperative pain to chronic pain: part 2 – limiting the transition. Trends Anaes Crit Care. 2014;4(2):71–75. doi:10.1016/j.tacc.2014.04.002.

[11] Burns S, Urman R, Pian R, Coppes OJM. Reducing new persistent opioid use after surgery: a review of interventions. Curr Pain Headache Rep. 2021;25(5):27. doi:10.1007/s11916-021-00943-6. 33760983 PMC7990836

[12] Poland F, Spalding N, Gregory S, McCulloch J, Sargen K, Vicary P. Developing patient education to enhance recovery after colorectal surgery through action research: a qualitative study. BMJ Open. 2017;7(6):e013498. doi:10.1136/bmjopen-2016-013498. PMC5577868 28667197

[13] Khorfan R, Shallcross ML, Yu B, Sanchez N, Parilla S, Coughlin JM, Johnson JK, Bilimoria KY, Stulberg JJ. Preoperative patient education and patient preparedness are associated with less postoperative use of opioids. Surgery. 2020;167(5):852–58. doi:10.1016/j.surg.2020.01.002. 32087946 PMC7192392

[14] Kruzik N. Benefits of preoperative education for adult elective surgery patients. AORN J. 2009;90(3):381–87. doi:10.1016/j.aorn.2009.06.022. 19735761

[15] Surgical safety in Canada: a 10-year review of CMPA and HIROC medico-legal data. [accessed 2023 Aug 2]. https://www.patientsafetyinstitute.ca:443/en/toolsResources/Surgical-Safety-in-Canada/Pages/default.aspx.

[16] Hill MV, Stucke RS, Billmeier SE, Kelly JL, Barth RJ. Guideline for discharge opioid prescriptions after inpatient general surgical procedures. J Am Coll Surg. 2018;226(6):996–1003. doi:10.1016/j.jamcollsurg.2017.10.012. 29198638

[17] Neuman MD, Bateman BT, Wunsch H. Inappropriate opioid prescription after surgery. Lancet. 2019;393(10180):1547–57. doi:10.1016/S0140-6736(19)30428-3. 30983590 PMC6556783

[18] Federal, provincial, and territorial Special Advisory Committee on the Epidemic of Opioid Overdoses. CDC guideline for prescribing opioids for chronic pain — United States, 2016. MMWR Recomm Rep. 2016;65. doi:10.15585/mmwr.rr6501e1er. 26987082

[19] Opioid- and stimulant-related harms — Canada.ca. [accessed 2023 Aug 2]. https://health-infobase.canada.ca/substance-related-harms/opioids-stimulants/.

[20] Canada H. Canada’s opioid crisis (fact sheet). Published 2020 Mar 19 [accessed 2023 Aug 2]. https://www.canada.ca/en/health-canada/services/publications/healthy-living/canada-opioid-crisis-fact-sheet.html.

[21] Canadian Institute for Health Information. Opioid prescribing in Canada: how are practices changing? Published online. 2019. https://www.cihi.ca/sites/default/files/document/opioid-prescribing-canada-trends-en-web.pdf.

[22] Wilton J, Abdia Y, Chong M, Karim ME, Wong S, MacInnes A, Balshaw R, Zhao B, Gomes T, Yu A, et al. Prescription opioid treatment for non-cancer pain and initiation of injection drug use: large retrospective cohort study. BMJ. 2021;375:e066965. doi:10.1136/bmj-2021-066965. 34794949 PMC8600402

[23] Smolina K, Crabtree A, Chong M, Zhao B, Park M, Mill C, Schütz CG. Patterns and history of prescription drug use among opioid-related drug overdose cases in British Columbia, Canada, 2015–2016. Drug Alcohol Depend. 2019;194:151–58. doi:10.1016/j.drugalcdep.2018.09.019. 30439611

[24] Clarke HA, Manoo V, Pearsall EA, Goel A, Feinberg A, Weinrib A, Chiu JC, Shah B, Ladak SSJ, Ward S, et al. Consensus statement for the prescription of pain medication at discharge after elective adult surgery. Can J Pain. 2020;4(1):67–85. doi:10.1080/24740527.2020.1724775. 33987487 PMC7951150

[25] Risk factors for opioid misuse, addiction, and overdose. DOL; [accessed 2023 Aug 2]. http://www.dol.gov/agencies/owcp/opioids/riskfactors.

[26] Clarke H, Azargive S, Montbriand J, Nicholls J, Sutherland A, Valeeva L, Boulis S, McMillan K, Ladak SSJ, Ladha K, et al. Opioid weaning and pain management in postsurgical patients at the Toronto General Hospital transitional pain service. Can J Pain. 2018;2(1):236–47. doi:10.1080/24740527.2018.1501669. 35005382 PMC8730554

[27] Busse JW, Craigie S, Juurlink DN, Buckley DN, Wang L, Couban RJ, Agoritsas T, Akl EA, Carrasco-Labra A, Cooper L, et al. Guideline for opioid therapy and chronic noncancer pain. CMAJ. 2017;189(18):E659–E666. doi:10.1503/cmaj.170363. 28483845 PMC5422149

[28] Ladha KS, Gagne JJ, Patorno E, Huybrechts KF, Rathmell JP, Wang SV, Bateman BT. Opioid overdose after surgical discharge. JAMA. 2018;320(5):502–04. doi:10.1001/jama.2018.6933. 30087999 PMC6142985

[29] Sawhney M, Watt-Watson J, McGillion M, Pain Education A. Intervention for patients undergoing ambulatory inguinal hernia repair: a randomized controlled trial. Can J Nurs Res. 2017;49(3):108–17. doi:10.1177/0844562117714704. 28841064

[30] Zohar-Bondar A, Stepan JG, Chapman T, Sacks H, Verrett I, Fufa DT. The effect of standardized perioperative patient education on opioid use after minor soft tissue procedures distal to the wrist. J Hand Surg Am. 2022;47(6):580.e1–580.e9. doi:10.1016/j.jhsa.2021.06.009. 34332818

[31] Rajput S, Tiwari T, Chaudhary A. Effect of preoperative multimedia based video information on perioperative anxiety and hemodynamic stability in patients undergoing surgery under spinal anesthesia. J Family Med Prim Care. 2021;10(1):237. doi:10.4103/jfmpc.jfmpc_1544_20. 34017733 PMC8132850

[32] Sterne JAC, Savović J, Page MJ, Elbers RG, Blencowe NS, Boutron I, Cates CJ, Cheng H-Y, Corbett MS, Eldridge SM, et al. RoB 2: a revised tool for assessing risk of bias in randomised trials. BMJ. 2019;366:l4898. doi:10.1136/bmj.l4898. 31462531

[33] Sterne JA, Hernán MA, Reeves BC, Savović J, Berkman ND, Viswanathan M, Henry D, Altman DG, Ansari MT, Boutron I, et al. ROBINS-I: a tool for assessing risk of bias in non-randomised studies of interventions. BMJ. 2016;355:i4919. doi:10.1136/bmj.i4919. 27733354 PMC5062054

[34] Fahimi K, Abbasi A, Zahedi M, Amanpour F, Ebrahimi H. The effects of multimedia education on postoperative delirium in patients undergoing coronary artery bypass graft: a randomized clinical trial. Nurs Crit Care. 2020;25(6):346–52. doi:10.1111/nicc.12473. 31532055

[35] Lai VKW, Ho KM, Wong WT, Leung P, Gomersall CD, Underwood MJ, Joynt GM, Lee A. Effect of preoperative education and ICU tour on patient and family satisfaction and anxiety in the intensive care unit after elective cardiac surgery: a randomised controlled trial. BMJ Qual Saf. 2021;30(3):228–35. doi:10.1136/bmjqs-2019-010667. 32321777

[36] Stepan JG, Sacks HA, Verret CI, Wessel LE, Kumar K, Fufa DT. Standardized perioperative patient education decreases opioid use after hand surgery: a randomized controlled trial. Plast Reconstr Surg. 2021;147(2):409–18. doi:10.1097/PRS.0000000000007574. 33235041

[37] Hoorntje A, Waterval-Witjes S, Koenraadt KLM, Kuijer PPFM, Blankevoort L, Kerkhoffs GMMJ, van Geenen RCI. Goal attainment scaling rehabilitation improves satisfaction with work activities for younger working patients after knee arthroplasty: results from the randomized controlled ACTION Trial. J Bone Joint Surg. 2020;102(16):1445–53. doi:10.2106/JBJS.19.01471. 32453116 PMC7508269

[38] Che YJ, Gao YL, Jing J, Kuang Y, Zhang M. Effects of an informational video about anesthesia on pre- and post-elective cesarean section anxiety and recovery: a randomized controlled trial. Med Sci Monit. 2020:26. doi:10.12659/MSM.920428. PMC7165245 32265432

[39] Yuzkat N, Soyalp C, Turk O, Keskin S, Gulhas N. Effects of showing the operating room on preoperative anxiety and hemodynamics among patients with hypertension: a randomized controlled trial. Clin Exp Hypertens. 2020;42(6):553–58. doi:10.1080/10641963.2020.1723619. 32009491

[40] Strøm J, Nielsen CV, Jørgensen LB, Andersen NT, Laursen M. A web-based platform to accommodate symptoms of anxiety and depression by featuring social interaction and animated information in patients undergoing lumbar spine fusion: a randomized clinical trial. Spine J. 2019;19(5):827–39. doi:10.1016/j.spinee.2018.11.011. 30500464

[41] Klaiber U, Stephan-Paulsen LM, Bruckner T, Müller G, Auer S, Farrenkopf I, Fink C, Dörr-Harim C, Diener MK, Büchler MW, et al. Impact of preoperative patient education on the prevention of postoperative complications after major visceral surgery: the cluster randomized controlled PEDUCAT trial. Trials. 2018;19(1):288. doi:10.1186/s13063-018-2676-6. 29793527 PMC5968532

[42] Eley VA, Searles T, Donovan K, Walters E. Effect of an anaesthesia information video on preoperative maternal anxiety and postoperative satisfaction in elective caesarean section: a prospective randomised trial. Anaesth Intensive Care. 2013;41(6):774–81. doi:10.1177/0310057X1304100613. 24180719

[43] Vincent S, Paskey T, Critchlow E, Mann E, Chapman T, Abboudi J, Jones C, Kirkpatrick W, Namdari S, Hammoud S, et al. Prospective randomized study examining preoperative opioid counseling on postoperative opioid consumption after upper extremity surgery. Hand (New York, N.Y). 2022;17(2):200–05. doi:10.1177/1558944720919936. 32432491 PMC8984704

[44] Korkmaz S, Iyigun E, Tastan S. An evaluation of the influence of web-based patient education on the anxiety and life quality of patients who have undergone mammaplasty: a randomized controlled study. J Canc Educ. 2020;35(5):912–22. doi:10.1007/s13187-019-01542-1. 31119709

[45] Cheesman Q, DeFrance M, Stenson J, Weekes D, Feldman J, Abboud J, Austin L. The effect of preoperative education on opioid consumption in patients undergoing arthroscopic rotator cuff repair: a prospective, randomized clinical trial—2-year follow-up. J Shoulder Elbow Surg. 2020;29(9):1743–50. doi:10.1016/j.jse.2020.04.036. 32815803

[46] Platto JF, Maarouf M, Hendricks A, Kurtzman DJ, Shi VY. Animated video consultation for reducing pre-operative anxiety in dermatologic surgery. Dermatol Online J. 2019;25(3). doi:10.5070/D3253043328. 30982298

[47] Lee CH, Liu JT, Lin SC, Hsu TY, Lin CY, Lin LY. Effects of educational intervention on state anxiety and pain in people undergoing spinal surgery: a randomized controlled trial. Pain Manage Nurs. 2018;19(2):163–71. doi:10.1016/j.pmn.2017.08.004. 29153299

[48] Billquist EJ, Michelfelder A, Brincat C, Brubaker L, Fitzgerald CM, Mueller ER. Pre-operative guided imagery in female pelvic medicine and reconstructive surgery: a randomized trial. Int Urogynecol J. 2018;29(8):1117–22. doi:10.1007/s00192-017-3443-z. 28884342

[49] Wongkietkachorn A, Wongkietkachorn N, Rhunsiri P. Preoperative needs-based education to reduce anxiety, increase satisfaction, and decrease time spent in day surgery: a randomized controlled trial. World J Surg. 2018;42(3):666–74. doi:10.1007/s00268-017-4207-0. 28875242

[50] Alter TH, Ilyas AM, Prospective Randomized A. Study analyzing preoperative opioid counseling in pain management after carpal tunnel release surgery. J Hand Surg Am. 2017;42(10):810–15. doi:10.1016/j.jhsa.2017.07.003. 28890331

[51] Van Eck CF, Toor A, Banffy MB, Gambardella RA. Web-based education prior to outpatient orthopaedic surgery enhances early patient satisfaction scores: a prospective randomized controlled study. Orthop J Sports Med. 2018;6(1):232596711775141. doi:10.1177/2325967117751418. PMC5788117 29399589

[52] Pereira L, Figueiredo-Braga M, Carvalho IP. Preoperative anxiety in ambulatory surgery: the impact of an empathic patient-centered approach on psychological and clinical outcomes. Patient Educ Couns. 2016;99(5):733–38. doi:10.1016/j.pec.2015.11.016. 26654958

[53] Kalogianni A, Almpani P, Vastardis L, Baltopoulos G, Charitos C, Brokalaki H. Can nurse-led preoperative education reduce anxiety and postoperative complications of patients undergoing cardiac surgery?. Eur J Cardiovasc Nurs. 2016;15(6):447–58. doi:10.1177/1474515115602678. 26304701

[54] Lin SY, Huang HA, Lin SC, Huang YT, Wang KY, Shi HY. The effect of an anaesthetic patient information video on perioperative anxiety: a randomised study. Eur J Anaesthesiol. 2016;33(2):134–39. doi:10.1097/EJA.0000000000000307. 26196527

[55] Schmidt M, Eckardt R, Scholtz K, Neuner B, von Dossow-Hanfstingl V, Sehouli J, Stief CG, Wernecke K-D, Spies CD. Patient empowerment improved perioperative quality of care in cancer patients aged ≥ 65 years – a randomized controlled trial. Bruns H, ed. PLoS ONE. 2015;10(9):e0137824. doi:10.1371/journal.pone.0137824. 26378939 PMC4574984

[56] Sugai DY, Deptula PL, Parsa AA, Parsa FD. The importance of communication in the management of postoperative pain. Public Health. 2013;72(6):180–184. PMC3689499 23795326

[57] Campagna S, Clari M, Delfino C, Rolfo M, Rizzo A, Berchialla P, Ferrero A. Impact of a preoperative video-based educational intervention on postoperative outcomes in elective major abdominal surgery: a randomized controlled trial. J Gastrointest Surg. 2020;24(10):2295–97. doi:10.1007/s11605-020-04667-7. 32524360

[58] Ilyas AM, Chapman T, Zmistowski B, Sandrowski K, Graham J, Hammoud S. The effect of preoperative opioid education on opioid consumption after outpatient orthopedic surgery: a prospective randomized trial. Orthopedics. 2021;44(2):123–27. doi:10.3928/01477447-20210201-07. 33561870

[59] Paskey T, Vincent S, Critchlow E, Mann E, Chapman T, Aland C, Dodson C, Emper W, Freedman KB, Pedowitz D, et al. Prospective randomized study evaluating the effects of preoperative opioid counseling on postoperative opioid use after outpatient lower extremity orthopaedic surgery. J Surg Orthop Adv. 2021 Jan 1;30(1):2–6. 33851905

[60] Peng F, Peng T, Yang Q, Liu M, Chen G, Wang M. Preoperative communication with anesthetists via anesthesia service platform (ASP) helps alleviate patients’ preoperative anxiety. Sci Rep. 2020;10(1):18708. doi:10.1038/s41598-020-74697-3. 33127967 PMC7603311

[61] Mousavi Malek N, Zakerimoghadam M, Esmaeili M, Kazemnejad A. Effects of nurse-led intervention on patients’ anxiety and sleep before coronary artery bypass grafting. Crit Care Nurs Q. 2018;41(2):161–69. doi:10.1097/CNQ.0000000000000195. 29494371

[62] Kiran U, Ladha S, Makhija N, Kapoor P, Choudhury M, Das S, Gharde P, Malik V, Airan B. The role of Rajyoga meditation for modulation of anxiety and serum cortisol in patients undergoing coronary artery bypass surgery: a prospective randomized control study. Ann Card Anaesth. 2017;20(2):158. doi:10.4103/aca.ACA_32_17. 28393774 PMC5408519

[63] Palmer JB, Lane D, Mayo D, Schluchter M, Leeming R. Effects of music therapy on anesthesia requirements and anxiety in women undergoing ambulatory breast surgery for cancer diagnosis and treatment: a randomized controlled trial. J Clin Oncol. 2015;33:3162–68. doi:10.1200/JCO.2014.59.6049. 26282640 PMC4979095

[64] Ahmed KJ, Pilling JD, Ahmed K, Buchan J. Effect of a patient-information video on the preoperative anxiety levels of cataract surgery patients. J Cataract Refract Surg. 2019;45(4):475–79. doi:10.1016/j.jcrs.2018.11.011. 30709627

[65] Clarke H, Soneji N, Ko DT, Yun L, Wijeysundera DN. Rates and risk factors for prolonged opioid use after major surgery: population based cohort study. BMJ. 2014;348:g1251. doi:10.1136/bmj.g1251. 24519537 PMC3921439

[66] International Narcotics Control Board. Narcotic drugs - estimated world requirements for 2023 - statistics for 2021. Published online. 2023. https://www.incb.org/documents/Narcotic-Drugs/Technical-Publications/2022/Narcotic_Drugs_Technical_Publication_2022.pdf.

[67] Understanding the opioid overdose epidemic | Opioids | CDC. Published 2022 Oct 7 [accessed 2023 Aug 4]. https://www.cdc.gov/opioids/basics/epidemic.html.

[68] Huang A, Azam A, Segal S, Pivovarov K, Katznelson G, Ladak SS, Mu A, Weinrib A, Katz J, Clarke H, et al. Chronic postsurgical pain and persistent opioid use following surgery: the need for a transitional pain service. Pain Manage. 2016;6(5):435–43. doi:10.2217/pmt-2016-0004. 27381204

